# Combined Metabolomic and Transcriptomic Analysis Identifies Metabolic Signatures and Networks Associated with Semen Quality in Geese

**DOI:** 10.3390/ani16172763

**Published:** 2026-09-02

**Authors:** Lei Zhang, Guobo Sun, Rui Zhu, Gansheng Zhang, Qingyi Zhou, Yue Wang, Xiujun Duan

**Affiliations:** 1 School of Modern Animal Husbandry, Jiangsu Agri-Animal Husbandry Vocational College, Taizhou 225300, China; 2 Jiangsu Key Laboratory for High-Tech Research and Development of Veterinary Biopharmaceuticals, Engineering Technology Research Center for Modern Animal Science and Novel Veterinary Pharmaceutic Development, Taizhou 225300, China

**Keywords:** metabolomics, goose, semen quality, integrated multi-omics

## Abstract

Semen quality is a critical determinant of male fertility in poultry, yet the testicular metabolic and molecular mechanisms underlying seminal traits in geese remain poorly understood. This study aimed to characterize the testicular metabolomic and transcriptomic profiles of Zhedong white geese with divergent semen quality, and to identify key regulatory pathways and candidate genes influencing semen quality. Untargeted metabolomics and RNA sequencing were performed on testicular tissues from geese with high (H) and low (L) semen quality. Serum hormone measurements revealed significantly increased testosterone (T) and decreased follicle-stimulating hormone (FSH) levels in the H group compared with the L group (*p* < 0.05). Metabolomic analysis identified 207 differential metabolites (DEMs), which were significantly enriched in glycosylphosphatidylinositol (GPI)-anchor biosynthesis, the citrate (TCA) cycle, and alanine, aspartate, and glutamate metabolism. Integrated transcriptomic and metabolomic analyses, combined with KEGG Markup Language (KGML) network analysis, revealed a candidate regulatory network involving potential metabolites (arachidonic acid, pyruvic acid, fumaric acid, malic acid and beta-alanine), signaling pathways (the citrate (TCA) cycle, arginine biosynthesis, ABC transporters, glycine/serine/threonine metabolism, and efferocytosis), and candidate genes (*PLCB1, PLCB2, NT5C1A, TAT, LOC106042968* and *ABCG8*). Notably, *PLCB1*, which was significantly up-regulated in the H group, emerged as a candidate gene of particular interest that may be implicated in the regulation of metabolic pathways underlying semen quality in ganders. These findings provide novel insights into the testicular metabolic and transcriptomic determinants of semen quality in geese, and implicate *PLCB1* and associated pathways as potential targets for improving male fertility in poultry.

## Simple Summary

Semen quality is a critical determinant of reproductive efficiency in poultry, yet its molecular regulation remains poorly understood. Here, we performed integrated metabolomic and transcriptomic profiling of testicular tissues from Zhedong white geese with high (H) group versus low (L) group to identify key metabolites and genes associated with semen quality. Serum hormone analysis revealed that H-group ganders had significantly higher testosterone (T) and lower follicle-stimulating hormone (FSH) levels than L-group ganders (*p* < 0.05). Metabolomic profiling identified 207 differentially expressed metabolites (DEMs), with 93 up-regulated and 114 down-regulated in H group. Three candidate metabolites, malic acid, beta-alanine, and sarcosine, were significantly reduced in the H group (*p* < 0.01), and were primarily enriched in the TCA cycle, GPI-anchor biosynthesis, and alanine/aspartate/glutamate metabolism pathways. Integrated transcriptomic analysis further identified *PLCB1* as a notable candidate gene for goose semen quality. Our findings provide new insights into the biological factors affecting semen quality in geese and may help guide future strategies to improve male fertility in poultry.

## 1. Introduction

The Zhedong White Goose is a valuable indigenous poultry genetic resource in China, renowned for its rapid early growth rate, strong tolerance to roughage feeding, early sexual maturity, delicious meat with excellent taste, robust environmental adaptability, and elegant white plumage [1]. Despite these desirable traits, the industrialization of Zhedong White Goose production faces significant bottlenecks rooted in reproductive inefficiencies—most notably, the species’ strong reproductive seasonality and low semen quality in unselected males. With only 32–35 eggs laid per year by female geese [2], combined with persistently low fertilization and hatching rates in commercial flocks, farmers are forced to maintain an artificially high male-to-female ratio to compensate for poor reproductive performance, significantly increasing feeding costs and hindering the economic benefits. Collectively, these reproductive limitations have become a major barrier to improving the scaling and economic viability of Zhedong White Goose production. In recent years, the transformation of goose farming from traditional free-range systems to modern intensive production has increased demand for artificial insemination (AI) technology [3]. Unlike natural mating, AI allows precise control of genetic contribution and reduces male requirements, making it critical for improving breeding efficiency and reducing costs [4]. This shift has brought waterfowl semen quality mechanisms to the forefront of poultry research, as optimizing AI success depends on the stable acquisition of high-quality goose semen.

Semen quality is a critical determinant of reproductive efficiency and production performance in male poultry, representing a key economic trait in breeding systems [5]. Studies show that the heritability of male reproductive phenotypes (e.g., semen quality) is generally significantly higher than that of female traits in livestock [6], highlighting the potential for rapid genetic improvement through selective breeding of males. Unfortunately, research on goose reproductive performance has long exhibited a clear gender bias: most studies focus on egg-laying performance in female geese [7], while systematic research on male goose reproductive physiology and regulatory mechanisms remains scarce. Hu et al. [8] applied transcriptomic and selective sweep analysis to the testes of Tianfu Meat Goose II ganders at three different stages (36, 47, and 66 weeks of age), and identified *EP300*, *RAB31,* and *PRKG1* might be associated with the semen quality in ganders. Through comparative transcriptomic analysis of the hypothalamus, pituitary, and testes of high- and low-sperm-motility Landes ganders, Liu et al. [9] screened and identified multiple candidate pathways, including steroid hormone biosynthesis and GnRH signaling, as potential regulators of gander sperm motility. Ren et al. [10] performed transcriptomic and metabolomic profiling on the epididymis from high and low sperm mobility Zi ganders, and revealed *CNDP1*-citric acid integration as a potential key regulator of sperm motility in geese. Although the above studies have conducted multi-omics investigations on semen quality in geese, integrated transcriptomic and metabolomic analysis of goose testicular tissues has not yet been reported. Systematically elucidating the physiological basis and molecular regulatory mechanisms of male goose reproductive system development and semen quality will fill critical knowledge gaps in avian reproductive biology. Accordingly, in this study, we performed integrated transcriptomic and metabolomic analyses of testicular tissues from Zhedong White ganders with divergent semen quality. Our aim was to identify key genes and metabolites associated with semen quality, thereby providing theoretical basis and candidate molecular markers for the genetic improvement of reproductive performance in ganders.

## 2. Materials and Methods

### 2.1. Experimental Animals, Semen Quality and Sampling

Semen was collected by dorso-abdominal massage. The ejaculate volume of each gander was measured using a 1 mL medical disposable syringe. An aliquot of fresh semen was diluted at a ratio of 1:4 (*v:v*) in physiological saline and then immediately evaluated for density and motility under the Olympus CX43 microscope (Evident Corporation, Tokyo, Japan) using the Sperm Vision^TM^ CASA software (SAR Research-edition, MiniTüb, Tiefenbach, Germany). For morphological assessment, semen smears were prepared and stained with Wright-Giemsa stain kit (Cat. No. G1021, Solarbio, Beijing, China), and the percentage of morphologically normal sperm was determined by examining at least 200 sperm per sample under the same microscope. According to the findings of our earlier independent study, ganders were classified into high (H) and low (L) sperm quality groups based on their SQF (Semen Quality Factor) [8,11] values according to the following equation: SQF = ejaculate semen volume (mL) × sperm density (×10^6^/mL) × live and morphologically normal sperm (%). Six individuals from each group were then randomly selected for subsequent analyses. The sample size (*n* = 6 per group) was based on pilot experiments and previous studies [4,8,9,10], which indicated sufficient biological representation to detect major phenotypic differences. All ganders were fasted for 12 h, weighed and euthanized by carbon dioxide inhalation followed by cervical dislocation. Testes samples were collected and immediately snap-frozen in liquid nitrogen, then stored at −80 °C. Blood samples were centrifuged at 800× *g* for 5 min, and the upper serum layer was aspirated and stored at −80 °C.

### 2.2. Sex Hormone Level Measurements

Serum samples were thawed at room temperature after being removed from −80 °C storage. The concentrations of Gonadotropin-Releasing Hormone (GnRH, CB10019-Go), testosterone (T, CB10025-Go), follicle-stimulating hormone (FSH, CB10018-Go), and luteinizing hormone (LH, CB10030-Go) in the serum were measured using commercial enzyme-linked immunosorbent assay (ELISA) kits (COIBO BIO, Shanghai, China) according to the manufacturer’s instruction.

### 2.3. Metabolite Extraction and Mass Spectrometry Detection

Each testis tissue (30 mg) was placed with two steel balls and 600 μL methanol-water (4:1, *v/v*) containing mixed internal standards. After pre-cooling at −40 °C for 2 min, the samples were ground at 45 Hz for 2 min, ultrasonicated in ice water for 10 min, and kept at −40 °C overnight. The extracts were centrifuged at 13,800 rpm for 20 min at 4 °C. The supernatant was then divided into two aliquots for liquid chromatography-tandem mass spectrometry (LC-MS/MS) and gas chromatography-mass spectrometry (GC-MS) analyses, respectively. Quality control (QC) samples were prepared by pooling equal volumes of each sample extract. The pooled QC samples were processed and analyzed using the same procedures as the test samples.

LC-MS/MS detection: A 150 μL aliquot of the supernatant was transferred into LC vials and stored at −80 °C until analysis. LC-MS/MS analysis was performed on an ACQUITY UPLC HSS T3 column (100 mm × 2.1 mm, 1.8 μm, Waters Corporation, Milford, MA, USA) at 45 °C with a flow rate of 0.35 mL/min and an injection volume of 4 μL. The mobile phases consisted of phase A (water with 0.1% formic acid) and phase B (acetonitrile). The gradient elution program was as follows: 0–2 min, 5% B; 2–4 min, 5–30% B; 4–8 min, 30–50% B; 8–10 min, 50–80% B; 10–14 min, 80–100% B; 14–15 min, 100% B; 15–15.1 min, 100–5% B; 15.1–16 min, 5% B. Mass spectrometry data were collected using a Waters ACQUITY UPLC I-Class Plus coupled to a Thermo QE HF high-resolution mass spectrometer (Thermo Fisher Scientific, Waltham, MA, USA). Data were acquired in ESI+ and ESI− modes over *m/z* 70–1050, with full MS resolution of 60,000 and MS/MS resolution of 15,000 (both at *m/z* 200). Data-dependent MS/MS was triggered on the top 10 precursors per cycle using stepped NCE of 10, 20, and 40 eV. Key source parameters: spray voltage, +3800 V (ESI+) and −3200 V (ESI−); capillary temperature, 320 °C; aux gas heater temperature, 350 °C; sheath gas, 35 Arb; aux gas, 8 Arb; S-lens RF level, 50.

GC-MS detection: Another 150 μL aliquot of the supernatant was transferred into glass vials and dried under vacuum. The dried residue was derivatized by adding 80 μL methoxyamine hydrochloride in pyridine (15 mg/mL) and incubating at 37 °C for 60 min with shaking. Subsequently, 50 μL BSTFA, 20 μL n-hexane, and 10 μL internal standard mixture (C8/C9/C10/C12/C14/C16/C18/C20/C22/C24) were added, and the reaction was carried out at 70 °C for 60 min. After cooling to room temperature for 30 min, the samples were analyzed by GC-MS. GC-MS analysis was conducted on a DB-5MS column (30 m × 0.25 mm × 0.25 μm, Agilent J&W Scientific, Folsom, CA, USA) with helium carrier gas (≥99.999%) at 1.0 mL/min. The injector temperature was 260 °C, and 1 μL was injected in splitless mode with a 5.2 min solvent delay. The oven temperature program was: 60 °C for 0.5 min, ramped to 125 °C at 8 °C/min, then to 210 °C at 8 °C/min, followed by 15 °C/min to 270 °C, and finally 20 °C/min to 305 °C (held 5 min). Mass spectrometry data were collected using an Agilent 8890-5977B gas chromatography-mass spectrometry system (Agilent Technologies, Inc., Santa Clara, CA, USA) using an electron ionization (EI) source operated at 70 eV, with ion source temperature set to 230 °C and quadrupole temperature maintained at 150 °C. Full-scan mode was employed over *m/z* 50–500, with data acquisition synchronized to chromatographic separation for comprehensive analyte detection.

The raw metabolomics data reported in this paper have been deposited in OMIX, China National Center for Bioinformation/Beijing Institute of Genomics, Chinese Academy of Sciences [12,13] under the accession number OMIX018321 (https://ngdc.cncb.ac.cn/omix/preview/0CcCXVgqG1).

### 2.4. Metabolomic Data Analysis

Data quality was controlled using internal standards and QC samples. Raw LC-MS/MS data were processed with XCMS v4.5.1 [14] for baseline filtering, peak detection, alignment, and integration, followed by RSD filtering (≤0.3), missing value imputation, zero replacement, log_2_-transformation, and score-based filtering (≥36). GC-MS data were processed for internal standard, noise, and column bleed, and derivatization peaks were removed, ion peaks with >50% missing values (zeros) per group were excluded, remaining missing values were replaced with half of the minimum value, and the data were log_2_-transformed. GC-MS data were then normalized using segmented internal standard normalization. Based on the above workflows, metabolite abundances were obtained as relative quantifications based on peak area integration and internal standard normalization. To obtain high-quality qualitative and quantitative results, we merged the raw data acquired from LC-MS/MS and GC-MS into a single comprehensive data matrix. This matrix contained all extractable features suitable for further analysis. After data merging, we performed redundancy removal and peak merging to consolidate duplicated or overlapping features. Compounds were then filtered based on their identification confidence score, which ranged from 0 to 100. Only compounds with a score ≥ 70 were retained for downstream analysis; those with a score < 70 were considered unreliable and removed from the dataset.

Principal component analysis (PCA) was first performed as an unsupervised method to visualize overall sample distribution and assess analytical stability. A full PCA model was constructed using all metabolic features, with the first two principal components (PC1 and PC2) selected for 2D score plot visualization based on their cumulative explained variance. Subsequently, orthogonal partial least squares discriminant analysis (OPLS-DA) was performed as a supervised method to maximize the separation between groups and identify differential metabolites. Both PCA and OPLS-DA were conducted on the OECloud platform (https://cloud.oebiotech.com). To validate the OPLS-DA models and exclude the possibility of overfitting, permutation tests (n = 200) were performed by randomly reassigning group labels to obtain R^2^ and Q^2^ values for each permuted model. The original model was considered valid when its R^2^ and Q^2^ exceeded all permuted values and the intercept of the Q^2^ regression line was below zero, indicating no overfitting. Given the modest sample size (*n* = 6 per group), OPLS-DA results were interpreted as exploratory and used primarily for visualization and hypothesis generation. Normality was assessed via skewness and kurtosis, and homogeneity of variance was evaluated using Levene’s test prior to *t*-test application. Differentially expressed metabolites (DEMs) were identified using criteria of *p* < 0.05, fold change (FC) ≥ 1.2 or ≤0.833. Importantly, no multiple-testing correction was applied to the DEM analysis; therefore, the results were considered exploratory and should be interpreted with caution due to the increased risk of false-positive findings. Pearson correlation analysis was performed on the relative abundance values of the top 30 DEMs (based on the smallest *p*-values); statistical significance was set at *p* < 0.05. Kyoto Encyclopedia of Genes and Genomes (KEGG [15]) pathway enrichment analysis was carried out based on the KEGG IDs of the DEMs, and chemical metabolomics annotation was performed using the FunMeta platform (https://cloud.oebiotech.com/#/lm/home).

### 2.5. Transcriptomic Data Analysis

Testicular tissues from the H and L groups were also subjected to RNA-seq. To obtain the transcriptomic data, total RNA was extracted from each testicular tissue using TRIzol^®^ reagent (Invitrogen, Carlsbad, CA, USA) following the manufacturer’s protocol. RNA concentration and integrity were assessed using a NanoDrop spectrophotometer and an Agilent 2100 Bioanalyzer (Agilent Technologies, Santa Clara, CA, USA). Samples with high quality (A260/A280 ratio 1.8–2.0 and RIN ≥ 7.0) were then sent to OE Biotech Co., Ltd. (Shanghai, China) for library construction and sequencing on the Illumina HiSeq 2500 platform. Raw sequencing data were screened with Fastp (v 0.22.0) to obtain the clean reads, which were then mapped to the Taihu goose T2T genome (GCF_040182565.1) using hisat2 (v 2.0.5) for the downstream analysis. Gene-level quantification was then performed using HTSeq-count (v 2.5.0) with the reference gene annotation file, and the reads count for each protein-coding gene in each sample was obtained. The resulting count matrix was used for subsequent differential expression analysis. Differentially expressed genes (DEGs) were then identified using the DESeq2 R package (v 1.38.3) [10], and the criteria of FDR (*q*-value) < 0.05, |log_2_FC| ≥ 0.58. KEGG pathway analyses were performed by clusterProfiler software (4.0) [16], and *p* < 0.05 was considered to be significantly enriched. To validate the RNA-seq results, 4 randomly selected DEGs were examined by qRT-PCR, which confirmed the technical reliability and accuracy of the RNA-Seq data. Total RNA was reverse-transcribed into cDNA using the SuperScript III First-Strand Synthesis SuperMix (Thermo Fisher, Nanjing, China). Primers were designed with Primer 5.0 (Supplementary Table S1), and qPCR was performed on a CFX96 Real-Time System (Bio-Rad, Hercules, CA, USA) following the protocol described by Hu et al. [8]. The relative mRNA expression levels of these DEGs were normalized to the geometric mean of Ct values from the two reference genes *GAPDH* and *β-ACTIN*, using the comparative Ct (2^−ΔΔCt^) method [8]. The raw RNA-seq data have been deposited in the Genome Sequence Archive (GSA) under accession number CRA030664 (https://ngdc.cncb.ac.cn/gsa/s/5eqI9829).

### 2.6. Integrated Metabolomic and Transcriptomic Analysis

To explore the integrated regulatory networks between transcriptomic and metabolomic datasets, Spearman correlation analysis was performed between the expression levels of the top 30 DEGs (based on the smallest *q*-values, *q* < 0.05) and top 30 DEMs (based on the smallest *p*-values, *p* < 0.05). Statistical significance was set at *p* < 0.05, and correlation coefficients with |r| > 0.9 were considered extremely strong correlations. From the correlation matrix, the top 20 gene–metabolite pairs with the smallest *p*-values (*p* < 0.05) in both omics datasets were further selected to construct a focused gene–metabolite correlation network. To identify the most relevant biological pathways shared by the two omics layers, Venn analysis was performed to screen the pathways that were significantly enriched in both datasets (*p* < 0.05) for further integrated interpretation. KGML [17] (KEGG Markup Language) network analysis was employed to parse gene–metabolite interactions from KEGG pathways, and construct a node-link network. Topological metrics (node degree) were calculated to identify key regulatory nodes, and the top five nodes with the highest connectivity degrees in each category (DEGs, DEMs, and pathways) were selected for visualization.

### 2.7. Statistical Analysis

All statistical analyses in this study were performed using SPSS, version 19.0 (IBM, Armonk, NY, USA). The results were expressed as mean ± standard deviation. Differences were assessed using the *t*-test. Values of *p* < 0.05 was statistically significant, and *p* < 0.01 was highly statistically significant, *p* < 0.001 was extremely statistically significant.

## 3. Results

### 3.1. Comparison of Sex Hormone Levels

**Figure 1:**
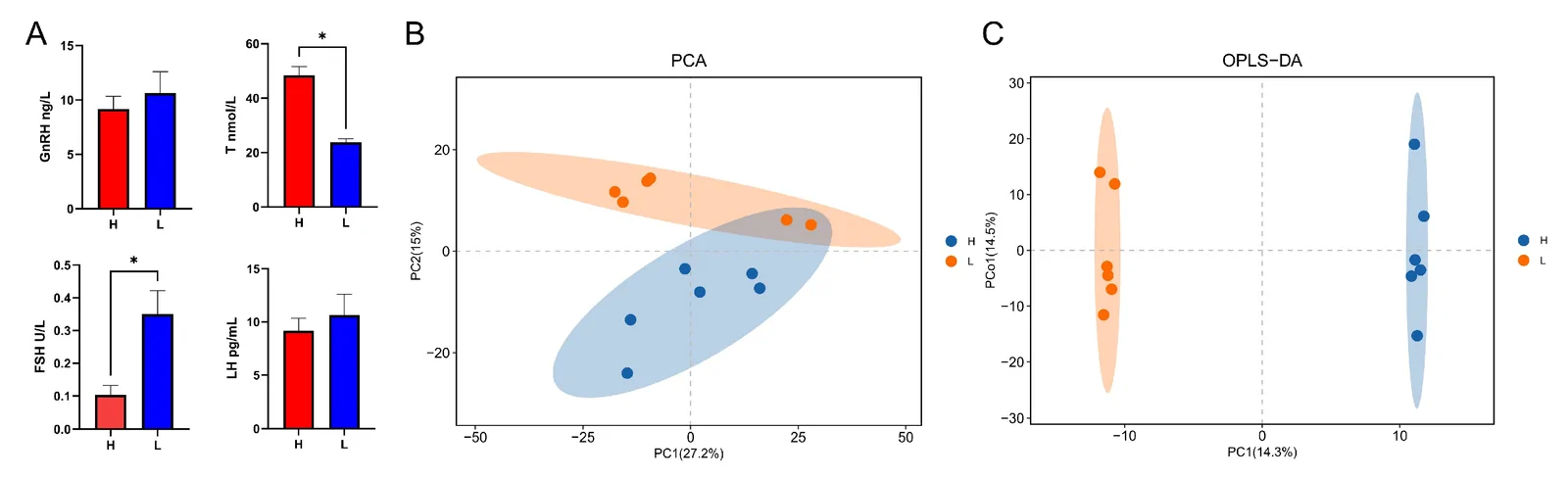
Hormonal and metabolomic comparisons between H and L groups. (**A**) Hormone level comparison between H and L groups, * represents statistically significant difference (*p* < 0.05); (**B**) PCA diagram of metabolites between H and L groups; each point in the figure represents a sample, different color–shape combinations denote distinct sample groups, and the ellipses indicate 95% confidence intervals; (**C**) OPLS-DA diagram of metabolites between H and L groups; each point in the figure represents a sample, with different color–shape combinations indicating different sample groups.

To explore the relationship between goose semen quality and hormones, the levels of GnRH, T, FSH and LH in blood serum were measured. As shown in Figure 1A, compared to the L group, serum T level was significantly increased in the H group (*p* < 0.05), whereas FSH level was significantly decreased (*p* < 0.05). However, no significant differences were detected in the serum levels of GnRH or LH between the two groups (*p* > 0.05).

### 3.2. Comparative Metabolomic Data Analysis

#### 3.2.1. PCA and OPLS-DA Analysis

PCA of QC samples (Supplementary Figure S1) revealed tight clustering, confirming robust analytical stability and reproducibility. This provided a solid foundation for subsequent downstream analyses. To characterize metabolic divergence between H and L groups, unsupervised PCA and OPLS-DA analysis were performed. PCA scores (Figure 1B) showed partial overlap between groups, with PC1 and PC2 explaining 27.2% and 16.0% of total variance, respectively. This pattern indicated both shared metabolic traits and inherent differences between the cohorts. In contrast, OPLS-DA analysis (Figure 1C) achieved complete separation along PC1 (14.5% variance explained), demonstrating strong discriminatory power. The clear group segregation in the supervised model underscores significant metabolomic disparities between H and L groups, providing a robust framework for subsequent identification of differential metabolic markers and mechanistic investigations. However, given the relatively modest sample size (*n* = 6 per group), these results should be interpreted with caution.

#### 3.2.2. Identification of DEMs

**Figure 2:**
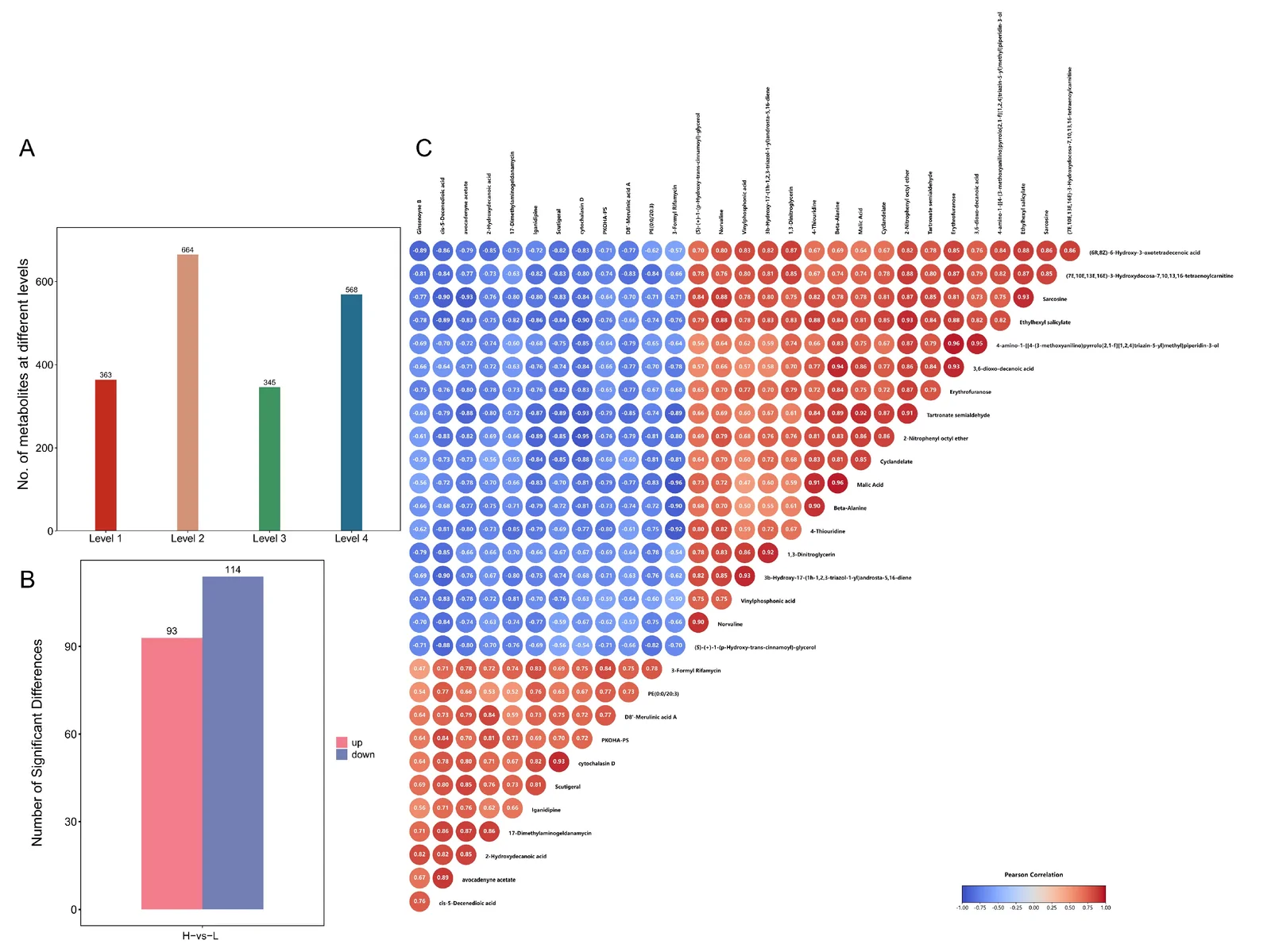
Comprehensive metabolomic analysis of testis between H and L groups. (**A**) Summary statistics of metabolite numbers at different identification confidence levels; Level 1, Score ≥ 85; Level 2, 80 ≤ Score < 85; Level 3, 70 ≤ Score < 80; and Level 4, Score < 70; (**B**) Overview of DEMs identified between H and L groups; (**C**) Correlation matrix of the top 30 significant DEMs; red indicates a positive correlation, while blue indicates a negative correlation.

Metabolomic profiling of all samples yielded a total of 1940 annotated metabolites (Figure 2A). Differential expression analysis identified 207 significant DEMs when comparing H and L, among which 93 metabolites were significantly up-regulated and 114 were significantly down-regulated (Figure 2B). To more intuitively visualize the relationships among samples and the expression differences in metabolites across different samples, the top 30 DEMs with the smallest *p*-values were screened. Correlation analysis was then performed on these top 30 significant DEMs, and the results are shown in Figure 2C. A total of 19 pairs of metabolites exhibited extremely strong correlations (|r| > 0.9), among which 14 pairs showed correlation coefficients > 0.9 and five pairs showed correlation coefficients < −0.9 (Supplementary Table S2).

#### 3.2.3. DEMs Functional Annotation

**Figure 3:**
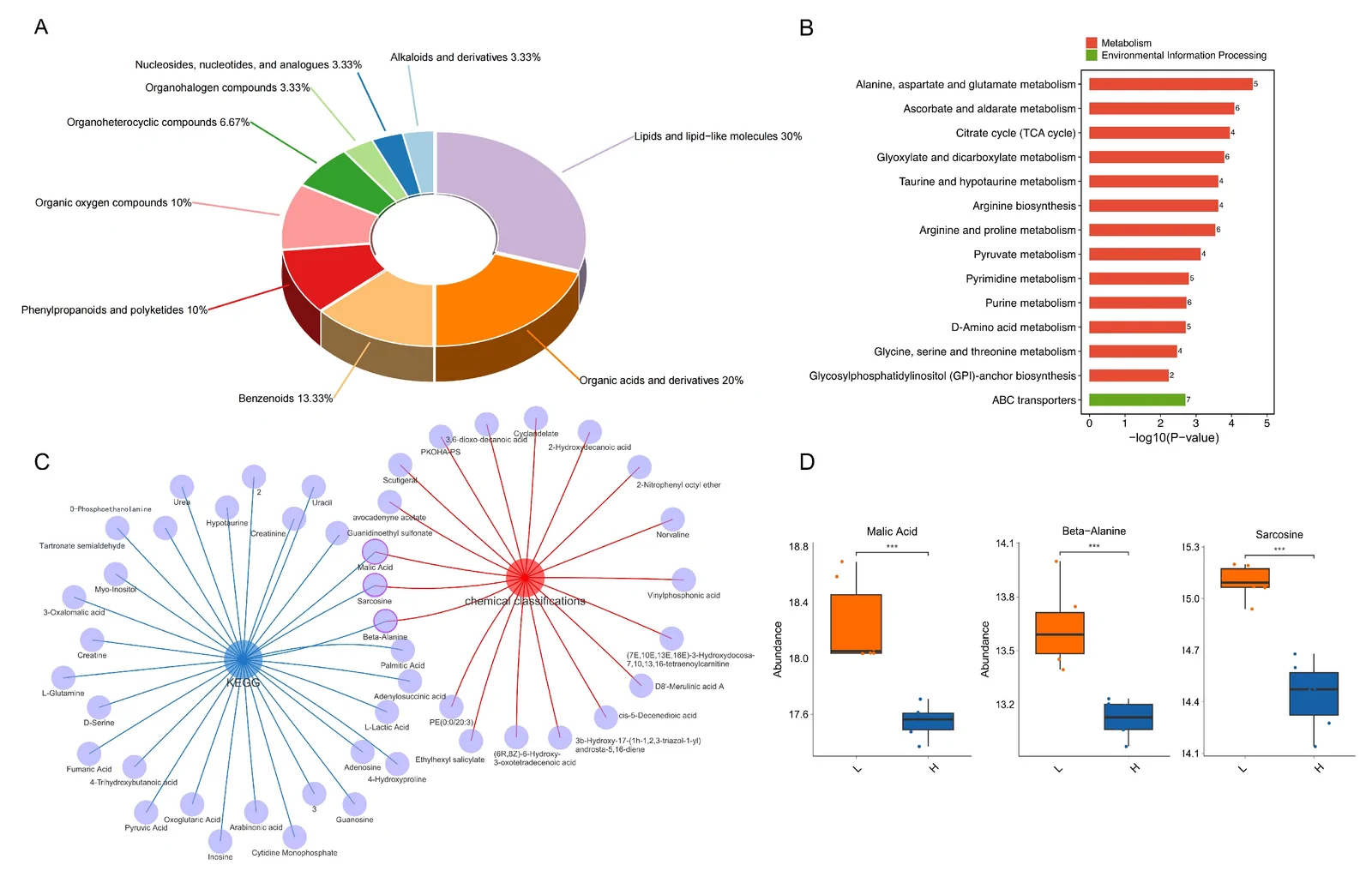
Comprehensive metabolomic analysis of testis between H and L groups. (**A**) Chemical classification of top 30 DEMs between H and L groups; (**B**) Top 15 KEGG enrichment analysis of metabolomic data between H and L groups; (**C**) Venn diagram of metabolites enriched in top 15 KEGG pathways and top 3 chemical classifications. Purple dots represent metabolites, blue lines indicate pathway associations, and red lines indicate chemical classification associations; (**D**) Box plot showing the relative abundance of malic acid, beta-alanine, and sarcosine in H and L groups, *** represents extremely statistically significant difference (*p* < 0.001).

Chemical metabolomics annotation of the top 30 significant DEMs revealed that these metabolites were primarily classified as lipids and lipid-like molecules, organic acids and derivatives, and benzenoids (Figure 3A). KEGG pathway enrichment analysis was performed to functionally annotate the DEMs. The top 15 pathways with the lowest *p*-values are presented as a bar chart in Figure 3B. These pathways were categorized into two major functional categories: Metabolism (14 pathways), Environmental Information Processing (1). Among the top 15 KEGG pathways, the three with the highest enrichment scores were glycosylphosphatidylinositol (GPI)-anchor biosynthesis (acyg00563), the citrate cycle (acyg00020), and alanine, aspartate, and glutamate metabolism (acyg00250), indicating that metabolic pathways, particularly those related to energy metabolism and amino acid metabolism, were predominantly affected. The intersection of metabolites significantly enriched the top 15 KEGG pathways, and the top three chemical classifications identified three shared metabolites (Figure 3C). Notably, the levels of malic acid, beta-alanine, and sarcosine were significantly lower in the H group than in the L group (*p* < 0.01).

### 3.3. Integrated Analysis of Transcriptome and Metabolome

**Figure 4:**
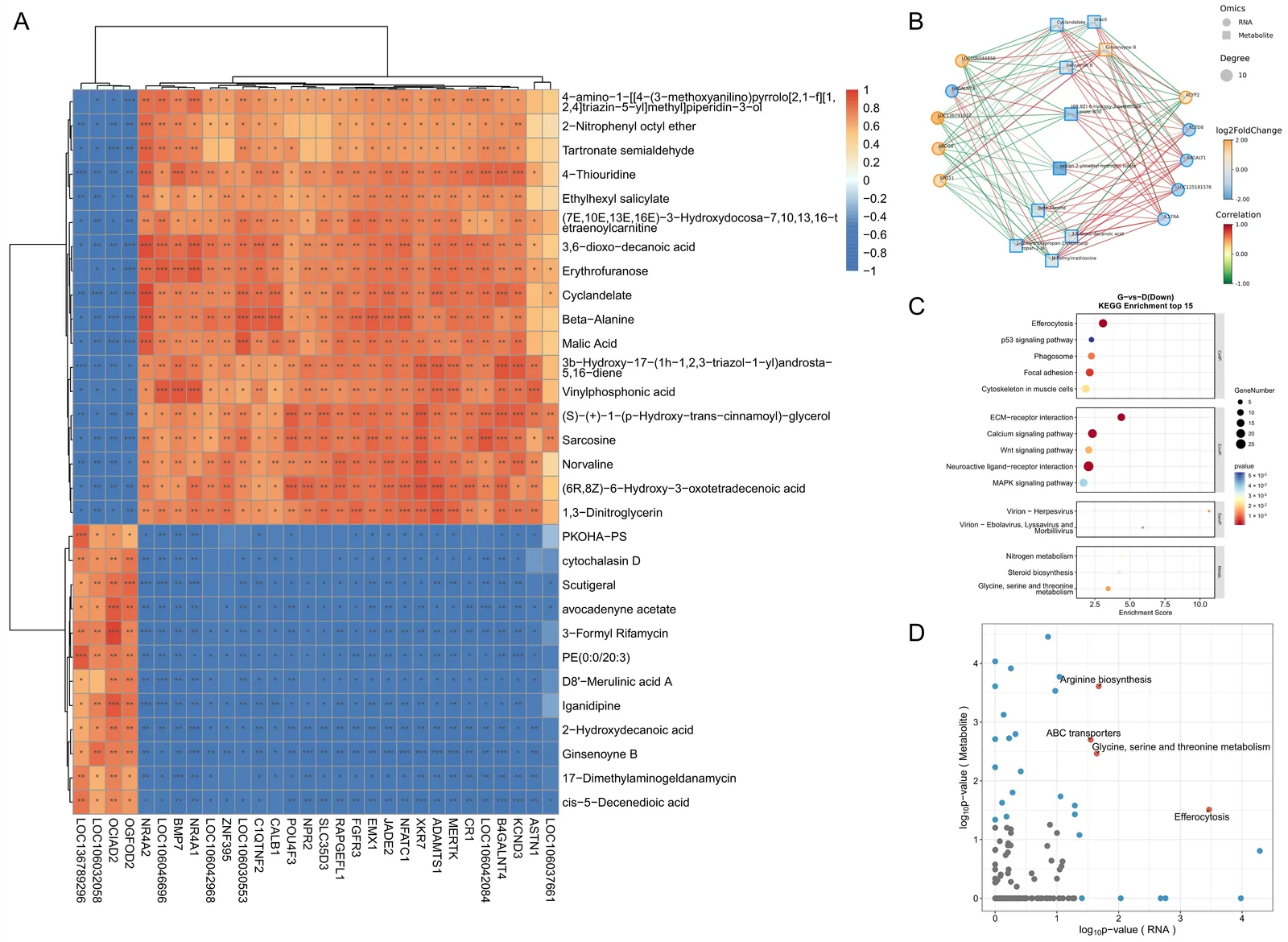
Integrated analysis of testicular transcriptome and metabolome between H and L groups. (**A**) Correlation heatmap of the top 30 DEMs and top 30 DEGs between H and L groups; orange indicates positive correlations, whereas blue indicates negative correlations, * represents statistically significant difference (*p* < 0.05), ** represents highly statistically significant difference (*p* < 0.01), *** represents extremely statistically significant difference (*p* < 0.001); (**B**) Top 20 entries with the most significant differences in both transcriptomics and metabolomics data between H and L groups, circular nodes denote transcriptomic data, while square nodes denote metabolomic data, with a gradient from red to blue indicating a shift from positive to negative log_2_FoldChange values; (**C**) Top 15 KEGG enrichment analysis of transcriptomics data between H and L groups; (**D**) Top 4 KEGG pathways significantly enriched in both transcriptomic and metabolomic data between H and L groups; gray indicates pathways that are not significant in either omics dataset, blue indicates pathways that are significant in only one omics dataset (*p* < 0.05), and red indicates pathways that are significant in both omics datasets (*p* < 0.05).

Comparative transcriptomic analysis of testicular tissues between H and L sperm quality groups identified 3334 DEGs, including 1552 up-regulated and 1782 down-regulated genes (Supplementary Figure S2). qRT-PCR was performed to validate the expression patterns of four randomly selected DEGs from the RNA-seq data. The qRT-PCR results showed consistent expression trends with the transcriptomic profiles, confirming the reliability and accuracy of the sequencing data (Supplementary Figure S3). Note that this validation only confirms the technical reliability of the RNA-seq data, but does not directly validate the functional roles of the following candidate genes. Based on the validated transcriptomic data, an integrative correlation analysis was performed to explore associations between metabolites and genes. To explore the potential association patterns between testicular metabolite profiles and gene expression patterns in geese, correlation analysis was performed using the top 30 DEMs and top 30 DEGs (Supplementary Table S3). As shown in Figure 4A, a substantial number of pairs showed significant correlations, suggesting potential coordinated regulatory changes between the transcriptomic and metabolomic layers. Importantly, these correlations represent exploratory associations only and do not imply direct causal regulatory relationships, particularly given the relatively small sample size (*n* = 6 per group). Based on the correlation analysis, the top 20 entries with the most significant differences in both transcriptomics and metabolomics were selected according to *p*-values, and a gene–metabolite correlation network was constructed (Figure 4B). The top 10 metabolites included beta-alanine, uracil, 3,6-dioxo-decanoic acid, among others, while the top 10 genes included *SPO11*, *ABCG8*, *B4GALNT4*, and so on. Transcriptome KEGG enrichment analysis revealed the top 15 significantly enriched pathways in the H vs. L comparison (Figure 4C). These pathways were classified into four functional categories: Cellular Processes (five pathways), Environmental Information Processing (five pathways), Genetic Information Processing (two pathways), and Metabolism (three pathways). Scatter plots of −log_10_(*p*-values) were generated to further visualize the significance of pathways across the two omics datasets. The top four potential core pathways were identified, including Arginine biosynthesis, ABC transporters, Glycine, serine and threonine metabolism, and Efferocytosis (Figure 4D).

**Figure 5:**
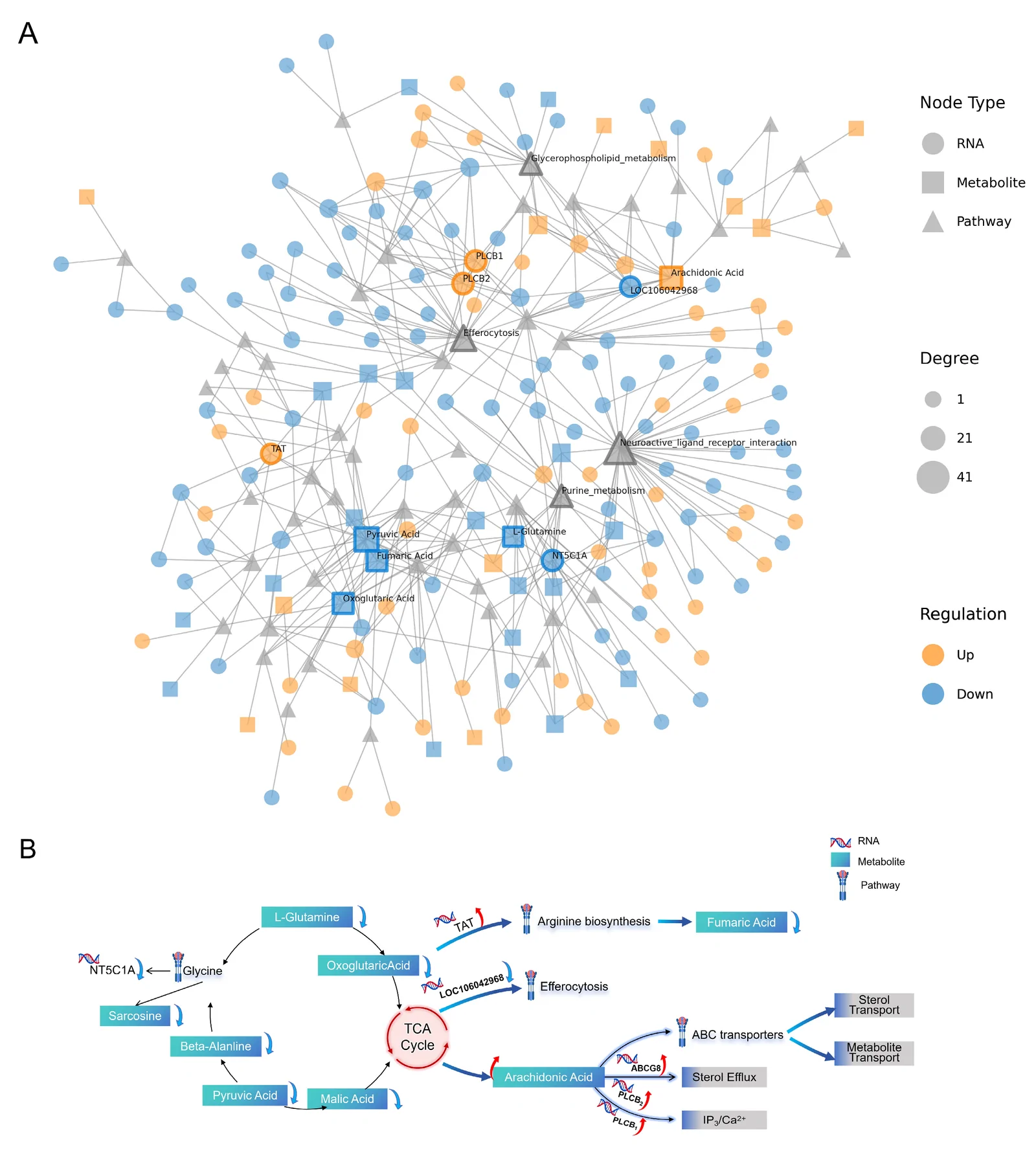
Construction of integrative networks for elucidating the molecular mechanisms underlying semen quality in ganders. (**A**) KGML network diagram; circular nodes represent DEGs, square nodes represent DEMs, and triangular nodes represent associated pathways; orange indicates positive correlations, whereas blue indicates negative correlations; this network is intended to highlight co-regulated modules and potential interactions between transcripts, metabolites, and pathways, rather than to infer direct causal mechanisms; (**B**) The hypothetical gene–pathway–metabolite network prioritizing candidate nodes associated with high semen quality in ganders, red arrows represent up-regulated expression, blue arrows represent down-regulated expression.

To further screen for differential metabolites associated with semen quality in ganders and to systematically investigate the interactive regulatory mechanisms between the transcriptome and metabolome, a KGML network analysis was performed based on the identified differential metabolites, key genes, and enriched signaling pathways. The top five nodes with the highest connectivity degrees in each category were selected, and the results are presented in Figure 5A. In the KGML network, the top five candidate genes included *PLCB1*, *PLCB2*, *TAT*, *LOC106042968*, and *NT5C1A*; the top five candidate metabolites comprised arachidonic acid, pyruvic acid, fumaric acid, oxoglutaric acid, and L-glutamine. For potential signaling pathways, since the Venn analysis identified only four KEGG pathways that were significantly enriched in both omics datasets (Figure 4D), these four co-enriched pathways were directly incorporated into the KGML network, including Arginine biosynthesis, ABC transporters, Glycine, serine and threonine metabolism, and Efferocytosis. Based on these findings, a hypothetical gene–pathway–metabolite network prioritizing candidate nodes associated with high semen quality in ganders was constructed in Figure 5B. It is noteworthy that, although the citrate cycle (TCA cycle) was not identified as a co-significant pathway between the transcriptomic and metabolomic datasets, it was nevertheless incorporated into the mechanistic diagram as a hypothetical central hub. Several key differential metabolites identified in this study, including oxoglutaric acid, malic acid, pyruvic acid, beta-alanine, sarcosine, L-glutamine, fumaric acid, and arachidonic acid, are directly involved in or closely connected to the TCA cycle. Given the exploratory nature of these analyses and the modest sample size, the KGML network should be interpreted as a hypothesis-generating framework rather than definitive proof of regulatory interactions.

## 4. Discussion

Metabolomics enables dynamic mapping of endogenous metabolite profiles and their responses to physiological or environmental changes [18,19]. Integrated multi-omics approaches further facilitate the elucidation of complex regulatory mechanisms underlying animal phenotypes [20]. The testis, as the core organ for spermatogenesis, supports germ cell differentiation and regulates the spermatogenic microenvironment via androgens such as T [21]. Recent evidence highlights that the local metabolic balance of lipids, amino acids, and nucleotides is critical for sperm energy supply, membrane stability, and antioxidant defense [22]. In avian species, sperm are already motile upon leaving the testis, suggesting that functional maturation depends more directly on the testicular metabolic microenvironment [23,24]. For example, carnitine-mediated fatty acid β-oxidation may enhance mitochondrial energy supply for sperm motility, while antioxidant metabolites like glutathione and vitamin E are positively correlated with sperm survival, and excessive polyunsaturated fatty acids may impair sperm function via lipid peroxidation [25,26,27]. Based on this, we used geese with divergent SQF as a model and employed untargeted metabolomics [28] combined with transcriptomics to investigate gene–metabolite regulatory mechanisms associated with semen quality.

In the present study, significant differences in serum sex hormone levels (T and FSH) were observed between the H and L groups. In parallel, metabolomic profiling of testicular tissues identified 207 DEMs between the two groups, suggesting that systemic hormonal changes are accompanied by distinct metabolic alterations at the testicular level. To elucidate the biological functions of these DEMs, KEGG pathway enrichment analysis was performed. The most significantly enriched pathways included GPI-anchor biosynthesis, the TCA cycle, and alanine, aspartate, and glutamate metabolism, all of which are closely linked to energy metabolism and cellular signaling. Among these, the TCA cycle serves as the primary ATP-generating pathway within mitochondria and plays a critical role in supporting spermatogenesis [29]. Notably, TCA cycle intermediates such as α-ketoglutarate function as essential cofactors for epigenetic modifiers that regulate histone modifications and m6A RNA methylation [30,31]. In a recent study, Ding et al. [32] reported testis-specific metabolite alterations in sterile cattle-yak hybrids and linked the TCA cycle intermediate α-ketoglutarate to the expression of meiotic genes, providing a useful reference for understanding the potential role of these metabolites in spermatogenesis across species. The alanine, aspartate, and glutamate metabolic pathway features three core amino acids that serve as important modulators of sperm function [33]. This pathway has been identified as significantly enriched in relation to differential sperm metabolites in bulls [34] and found to be disrupted in patients with non-obstructive azoospermia [35]. Of particular relevance to our study, Ren et al. [10] integrated transcriptomic and metabolomic analyses in Zi geese with divergent sperm motility and identified alanine, aspartate, and glutamate metabolism as a critical pathway influencing semen quality in ganders. Together, these findings suggest that the TCA cycle and alanine/aspartate/glutamate metabolism may play important roles in influencing semen quality in ganders, though validation in larger cohorts is needed given the limited sample size (*n* = 6 per group).

Based on metabolic potential critical pathways, three metabolites, including malic acid, beta-alanine, and sarcosine, were identified, all of which exhibited significantly lower abundance levels in the H group compared to the L group (*p* < 0.05). Given their well-documented roles in energy metabolism and reproductive function [36,37,38], we focused our subsequent analysis on the potential effects of malic acid and beta-alanine on semen quality in ganders. Malic acid, a key intermediate in the TCA cycle, plays an essential role in mitochondrial ATP production, which provides energy for sperm motility. In addition, malic acid may enhance the body’s antioxidant capacity by increasing the expression of antioxidant enzymes such as SOD and GSH-Px, thereby protecting testicular tissue from oxidative damage and maintaining the health of germ cells [36]. Notably, a significant reduction in malic acid levels was observed in the seminal plasma of patients with asthenozoospermia, suggesting its association with impaired sperm motility [37]. In bovine testicular tissue, L-malic acid was identified as one of the specifically elevated metabolites in infertile dzo (cattle-yak hybrids), positioning it as a key TCA cycle intermediate linked to male infertility [29]. Beta-alanine, together with histidine, forms the naturally occurring dipeptide carnosine, which has been reported to improve sperm quality during in vitro liquid storage. Sarkar et al. [39] detected beta-alanine in the male reproductive tract of the Japanese quail, with concentrations of 54.06 μM in testicular fluid, 109.67 μM in seminal plasma and 51.03 μM in cloacal gland secretion, indicating that beta-alanine is a naturally abundant metabolite in the male reproductive tract and may participate in reproductive regulation through dipeptide carnosine. In addition to serving as a carnosine precursor, beta-alanine also acts as a TCA cycle-related intermediate and mainly exerts its effects through modulating taurine function. Although metabolomic studies on beta-alanine in relation to semen quality remain limited, the potential biological effects of beta-alanine supplementation have been well documented. For instance, beta-alanine competitively inhibits taurine transport, which has been shown to reduce serum testosterone and LH levels and sperm motility in rats [38]; it could also inhibit Na^+^, K^+^-ATPase in hamster sperm [40] and reduce taurine uptake in mouse Sertoli cells [41]. Collectively, these literature-based findings, combined with our observation of reduced beta-alanine and malic acid levels in the H group, support the hypothesis that both metabolites may serve as potential metabolites influencing semen quality in ganders, although direct evidence in geese remains to be established.

Through integrated transcriptomic and metabolomic profiling, we performed a KGML-based network analysis incorporating differential metabolites, key genes, and enriched signaling pathways. On this basis, we constructed a proposed gene–pathway–metabolite network prioritizing candidate nodes potentially associated with semen quality in ganders. The network involved potential key metabolites (including arachidonic acid, pyruvic acid, fumaric acid, malic acid and beta-alanine), signaling pathways (TCA cycle, arginine biosynthesis, ABC transporters, glycine/serine/threonine metabolism, and efferocytosis), and genes (*PLCB1*, *PLCB2*, *NT5C1A*, *TAT*, *LOC106042968* and *ABCG8*). In light of previous findings linking *PLCB1* to spermatogenesis, meiosis, and male infertility [42,43,44], we prioritized *PLCB1* (phospholipase C beta 1) as the candidate gene of interest. *PLCB1* hydrolyzes phosphatidylinositol 4,5-bisphosphate (PIP2) to produce inositol 1,4,5-trisphosphate (IP3), which then activates IP3 receptors on the endoplasmic reticulum to release Ca^2+^ from intracellular stores, generating the calcium oscillations essential for oocyte activation during fertilization [42]. *PLCB1* has been implicated in meiosis, DNA repair, and folliculogenesis, and is considered a candidate gene for premature ovarian failure and ovarian dysfunction, with additional links to male infertility [43]. Notably, PLCB1 protein expression in the human testis is highly restricted to preleptotene spermatocytes and spermatogonia cells [44]. In rats, comparative transcriptomic analyses of pachytene spermatocytes, round spermatids, and mature spermatozoa revealed that *PLCB1* is strongly associated with meiotic progression [45]. Tang et al. [46] conducted transcriptomic comparisons of the hypothalamic–pituitary–gonadal (HPG) axis between ganders with normal and abnormal external genitals, identifying *PLCB1* as a potential key gene regulating external genitalia development in ganders. Our study further revealed significantly elevated *PLCB1* mRNA transcripts in the H group relative to the L group. *PLCB1* also indirectly modulates arachidonic acid metabolism, influencing sterol and metabolite transport essential for spermatogenesis and androgen synthesis [47]. Collectively, these findings suggest that *PLCB1* may play a critical regulatory role in modulating semen quality in geese.

While our findings provide novel insights into the metabolic regulation of semen quality in geese, several limitations should be acknowledged. First, this study was conducted with a single breed of geese raised under specific management conditions, which may limit the generalizability of our results to other breeds or production systems with distinct genetic backgrounds, nutritional requirements, or environmental constraints. Second, the modest sample size (*n* = 6 per group) may constrain the statistical power and robustness of our findings, particularly for multi-omics integrative analyses where complex interactions between genes, metabolites, and pathways are explored. Future studies with larger cohorts are warranted to confirm the reproducibility of our results and enhance the reliability of statistical inferences. Third, the candidate genes identified in this study (*PLCB1*, *PLCB2*, *NT5C1A*, *TAT*, *LOC106042968* and *ABCG8*) were solely derived from RNA-seq analysis, and their functional roles in regulating taurine and beta-alanine metabolism remain to be validated. Subsequent studies incorporating qRT-PCR, Western blotting, and functional assays such as gene knockdown or over-expression are essential to validate the functions of these candidate genes and to establish the causal relationships between these genes and metabolic phenotypes. Fourth, our discussion of taurine-related mechanisms underlying beta-alanine effects was primarily based on literature from other species, including rodents and humans. Finally, our data reveal correlative relationships among testicular transcriptomic and metabolic alterations and semen quality, but do not establish causal mechanisms. The co-expression network we constructed should be interpreted as a hypothesis-generating framework rather than definitive proof of regulatory interactions. Future mechanistic studies combining targeted metabolomics, transcriptomics, and functional validation are required to elucidate the precise molecular pathways linking testicular transcriptomic and metabolic regulation to improved reproductive performance in geese.

## 5. Conclusions

In conclusion, despite its limitations, this study provides a multi-omics resource for understanding the metabolic and transcriptional regulation of semen quality in geese. Key findings include the enrichment of the TCA cycle and alanine/aspartate/glutamate metabolism, reduced levels of malic acid, beta-alanine, and sarcosine in the H group, and the prioritization of *PLCB1* as a notable candidate gene. These results offer promising targets for future functional validation and provide foundational data for improving reproductive performance in poultry.

## Data Availability

The original data presented in the study are openly available in National Genomics Data Center repository under the accession number OMIX018321 and CRA030664.

## Supplementary Materials

The following supporting information can be downloaded at: https://www.mdpi.com/article/10.3390/ani16172763/s1, Figure S1: PCA of QC samples; Figure S2: Transcriptomic identification of DEGs in testicular tissues between H and L groups; Figure S3: qRT-PCR validation for RNA-Seq; Table S1: Primer information for qRT-PCR. Table S2: Metabolite pairs with extremely strong correlations (|r| > 0.9). Table S3: Top 30 DEGs in the testicular transcriptome between H and L groups.
