# Supplementary material for: Combined Metabolomic and Transcriptomic Analysis Identifies Metabolic Signatures and Networks Associated with Semen Quality in Geese
Source: Animals (Basel). 2026 Sep 2;16(17):2763. doi: 10.3390/ani16172763 (PMC13565586; doi:10.3390/ani16172763)
Supplement: Supplementary file 1 [file animals-16-02763-s001.zip › Table S1 Primer information for qRT-PCR.pdf]

Table S1. Primer information for qRT-PCR.

Gene	Sequence (5′-3′)	Tm (°C)
CYP17A1	F: CGACGAGAAGGAGTGGGACAAG<br>R: GCGAGGGCGAGTGGATGTG	59
INSL3	F: GCAGCCTTCGCCGACCTC<br>R: GGAGCAGCAGGAACCAGCAG	60
WNT3A	F: CTGTGCCAGTTTTCTCACCAT<br>R: AAGGCAAAGTCATTCACAACAA	58
MYCBP	F: GCTACACCGAGTCCTTCTCAGATAAG<br>R: TGTCCGACTGCTCAAAGTTCTCC	57
GADPH	F: GTGAAAGTCGGAGTCAACGG<br>R: CTTGGACTTTGCCAGACAGGA	60
β-ACTIN	F: TATTGCTGCGCTCGTTGTTG<br>R:ACGATGGATGGGAAGACAGC	60
