# Supplementary material for: Combined Metabolomic and Transcriptomic Analysis Identifies Metabolic Signatures and Networks Associated with Semen Quality in Geese
Source: Animals (Basel). 2026 Sep 2;16(17):2763. doi: 10.3390/ani16172763 (PMC13565586; doi:10.3390/ani16172763)
Supplement: Supplementary file 1 [file animals-16-02763-s001.zip › Table S2 Metabolite pairs with extremely strong correlations (r 0.9).pdf]

Table S2. Metabolite pairs with extremely strong correlations (|r| > 0.9).

Metabolite_1	Metabolite_2	Correlation
Malic Acid	Beta-Alanine	0.96
4-amino-1-[[4-(3-methoxyanilino)pyrrolo[2,1-f][1,2,4]triazin-5-yl]methyl]piperidin-3-ol	Erythrofuranose	0.96
4-amino-1-[[4-(3-methoxyanilino)pyrrolo[2,1-f][1,2,4]triazin-5-yl]methyl]piperidin-3-ol	3,6-dioxo-decanoic acid	0.95
3,6-dioxo-decanoic acid	Beta-Alanine	0.94
3,6-dioxo-decanoic acid	Erythrofuranose	0.93
Vinylphosphonic acid	3b-Hydroxy-17-(1h-1,2,3-triazol-1-yl)androsta-5,16-diene	0.93
cytochalasin D	Scutigeral	0.93
Sarcosine	Ethylhexyl salicylate	0.93
2-Nitrophenyl octyl ether	Ethylhexyl salicylate	0.93
1,3-Dinitroglycerin	3b-Hydroxy-17-(1h-1,2,3-triazol-1-yl)androsta-5,16-diene	0.92
Malic Acid	Tartronate semialdehyde	0.92
Malic Acid	4-Thiouridine	0.91
Tartronate semialdehyde	2-Nitrophenyl octyl ether	0.91
Beta-Alanine	4-Thiouridine	0.90
3-Formyl Rifamycin	4-Thiouridine	-0.92
avocadenyne acetate	Sarcosine	-0.93
cytochalasin D	Tartronate semialdehyde	-0.93
cytochalasin D	2-Nitrophenyl octyl ether	-0.95
3-Formyl Rifamycin	Malic Acid	-0.96
