# Supplementary material for: Combined Metabolomic and Transcriptomic Analysis Identifies Metabolic Signatures and Networks Associated with Semen Quality in Geese
Source: Animals (Basel). 2026 Sep 2;16(17):2763. doi: 10.3390/ani16172763 (PMC13565586; doi:10.3390/ani16172763)
Supplement: Supplementary file 1 [file animals-16-02763-s001.zip › Table S3 Top 30 DEGs screend from RNA-Seq.pdf]

Table S3. Top 30 DEGs in the testicular transcriptome between H and L groups.

gene_id	log_2FC	Regulation	description
LOC106046696	-4.50	Down	TBC1 domain family member 24-like
NR4A2	-2.08	Down	nuclear receptor subfamily 4 group A member 2
KCND3	-1.81	Down	potassium voltage-gated channel subfamily D member 3
ADAMTS1	-1.42	Down	ADAM metallopeptidase with thrombospondin type 1 motif 1
OGFOD2	1.09	Up	2-oxoglutarate and iron dependent oxygenase domain containing 2
LOC106032058	2.34	Up	uncharacterized LOC106032058
NR4A1	-1.82	Down	nuclear receptor subfamily 4 group A member 1
SLC35D3	-1.91	Down	solute carrier family 35 member D3
OCIAD2	1.77	Up	OCIA domain containing 2
B4GALNT4	-1.36	Down	beta-1%2C4-N-acetyl-galactosaminyltransferase 4
LOC106030553	-1.35	Down	carbonic anhydrase 13-like
LOC106037661	-3.02	Down	glycine receptor subunit alpha-4
LOC106042968	-1.92	Down	cytosolic phospholipase A2 epsilon-like
LOC136789296	1.12	Up	calpain-1 catalytic subunit
POU4F3	-2.01	Down	POU class 4 homeobox 3
RAPGEFL1	-1.89	Down	Rap guanine nucleotide exchange factor like 1
XKR7	-2.15	Down	XK related 7
BMP7	-2.57	Down	bone morphogenetic protein 7
FGFR3	-1.40	Down	fibroblast growth factor receptor 3
EMX1	-1.61	Down	empty spiracles homeobox 1
NFATC1	-1.15	Down	nuclear factor of activated T cells 1
C1QTNF2	-1.59	Down	C1q and TNF related 2
NPR2	-1.56	Down	natriuretic peptide receptor 2
ZNF395	-1.06	Down	zinc finger protein 395
CALB1	-1.56	Down	calbindin 1
JADE2	-1.01	Down	jade family PHD finger 2
LOC106042084	-1.65	Down	betaine--homocysteine S-methyltransferase 1
CR1	-1.18	Down	complement C3b/C4b receptor 1 (Knops blood group)
ASTN1	-1.81	Down	astrotactin 1
MERTK	-1.12	Down	MER proto-oncogene%2C tyrosine kinase
